# The predictive value of neutrophil-to-lymphocyte ratio in the efficacy of percutaneous lumbar disc radiofrequency ablation for lumbar disc herniation

**DOI:** 10.3389/fpain.2025.1543317

**Published:** 2025-05-29

**Authors:** Xin Zhao, Jialei Zhang, Jing Li, Kai Cao, Jie Wu

**Affiliations:** Department of Pain Treatment, Changzhi People’s Hospital Affiliated to Changzhi Medical College, Changzhi, China

**Keywords:** neutrophil, lymphocyte, lumbar disc herniation, percutaneous intradiscal radiofrequency thermocoagulation, pain

## Abstract

**Objective:**

To explore the predictive value of the Neutrophil-to-Lymphocyte Ratio (NLR) in the prognosis of patients with Lumbar Disc Herniation (LDH) undergoing Percutaneous Intradiscal Radiofrequency Thermocoagulation (PIRFT).

**Methods:**

A total of 121 patients with LDH undergoing PIRFT treatment were selected, ranging in age from 35 to 65 years old, with no gender restrictions. Blood samples were collected in the morning after admission while fasting, and the absolute neutrophil and lymphocyte counts in the blood were detected using the enzyme-linked immunosorbent assay (ELISA) method to calculate the Neutrophil-to-Lymphocyte Ratio (NLR). Patients were divided into two groups according to the modified Macnab criteria: the Effective group (E group) and the Invalid group (I group). The Visual Analogue Scale (VAS), Oswestry Disability Index (ODI), and Japanese Orthopaedic Association (JOA) scores were used to assess the pain level and activity ability of the patients before treatment and at 90 days and 180 days post-treatment. The correlation between NLR and ODI, JOA scores was analyzed using rank correlation analysis, and the predictive value of NLR for the therapeutic effect of PIRFT was analyzed using the Receiver Operating Characteristic (ROC) curve.

**Results:**

A total of 121 patients were ultimately enrolled. Based on the follow-up results at 90 days post-surgery, there were 110 cases in the effective group (E group) and 11 in the ineffective group (I group). The results showed that before treatment, the NLR levels in the E group were significantly lower than those in the I group (*P* < 0.05), and there were no significant differences in ODI and JOA scores between the two groups (*P* > 0.05). Ninety days after treatment, the NLR levels in the I group remained significantly higher than those in the E group (*P* < 0.05), and the E group's ODI and JOA scores showed significant improvement compared to before treatment (*P* < 0.05). In contrast, the I group only showed improvement in ODI scores (*P* < 0.05), with no significant change in JOA scores (*P* > 0.05). Additionally, the I group's ODI scores were significantly higher than those of the E group (*P* < 0.05), and their JOA scores were significantly lower than those of the E group (*P* < 0.05). All patients in the I group underwent a second radiofrequency ablation treatment. A comparison was made again after 180 days of treatment, and there were no significant differences in NLR levels between the two groups (*P* > 0.05). Both groups showed improvement in ODI and JOA scores compared to before treatment (*P* < 0.05), with no significant differences between the groups (*P* > 0.05). Rank correlation analysis showed that preoperative NLR levels were positively correlated with ODI scores at 90 days after PIRFT treatment (*r* = 0.386, *P* < 0.01) and negatively correlated with JOA scores (*r* = −0.326, *P* = 0.003). The results of the ROC curve analysis showed that the area under the ROC curve (AUC) was 0.803, the optimal diagnostic cutoff point was 1.975, the correct diagnosis index was 0.518, 95% CI: 0.650-0.955, *P* < 0.001.

**Conclusion:**

Preoperative inflammatory levels are one of the factors affecting the treatment of lumbar disc herniation with percutaneous radiofrequency ablation, and the Neutrophil-to-Lymphocyte Ratio (NLR) helps to predict the effectiveness of percutaneous disc radiofrequency ablation in treating lumbar disc herniation.

## Introduction

Lumbar disc herniation (LDH), as a common disease in orthopedics and pain management, is more prevalent among the middle-aged and elderly population. With the intensification of population aging and changes in daily lifestyles, there has been an increasing trend in the incidence of LDH and a tendency towards younger patients (1). Traditionally, LDH has been treated with surgical methods, which have the disadvantages of significant trauma, long recovery times, and a high recurrence rate. Conservative treatments, while having fewer adverse reactions, involve a long treatment period, and pain symptoms cannot be controlled in the short term, with the condition being prone to relapses (2).

Percutaneous intradiscal radiofrequency thermocoagulation (PIRFT) is one of the minimally invasive surgical methods currently used in pain clinics for the treatment of lumbar disc herniation (LDH). This technique involves the use of a specially designed electrode needle to emit radiofrequency currents, which generate high temperatures in the local tissue, thereby causing the local nucleus pulposus tissue to retract and reducing its physical compression on the nerve roots (3), achieving therapeutic effects. PIRFT is favored for its simplicity in operation (4), minimal trauma, and no risk of thermal damage to normal tissues (5). It is particularly suitable for patients with mild to moderate LDH who have not responded to conservative treatment and who do not require or are unwilling to undergo open surgery. It has shown significant effects in improving the symptoms and quality of life for such patients (6).

Inflammatory responses play a crucial role in the occurrence and development of lumbar disc herniation (LDH) and are almost present throughout the entire course of the disease (7, 8). However, it has not been proven whether there is a correlation between the preoperative inflammatory levels of patients and the therapeutic effects of PIRFT. Studies have shown that the neutrophil-to-lymphocyte ratio (NLR) holds significant value in the prognostic assessment and therapeutic monitoring of inflammatory diseases (9). The aim of this study is to conduct a correlation analysis between preoperative NLR and the prognosis of LDH treated with PIRFT, in hopes of further improving the therapeutic effects of PIRFT and exploring its possible mechanisms of action.

## Experimental procedures

### Study design

This study is a single-center prospective cohort. By analyzing the pre-treatment Neutrophil-to-Lymphocyte Ratio (NLR) levels in patients with lumbar disc herniation (LDH) and their post-treatment outcomes following Percutaneous Intradiscal Radiofrequency Thermocoagulation (PIRFT), we aim to explore the correlation between these two factors. This clinical trial has been approved by the Medical Ethics Committee of Changzhi City People's Hospital (Approval Number: 2024K020), all experiments were conducted in accordance with relevant guidelines and regulations, and it is registered with the Chinese Clinical Trial Registration Center (Registration Number: ChiCTR2400088475). All participating patients have signed informed consent forms.

### Patients

Patients with lumbar disc herniation (LDH) who underwent Percutaneous Intradiscal Radiofrequency Thermocoagulation (PIRFT) treatment in Pain Department from December 2023 to June 2024 were selected. Inclusion criteria: ages 35–65, no gender restrictions; clear clinical manifestations and imaging examinations confirming the diagnosis of lumbar disc herniation, with a single segment involved; positive foraminal compression test and straight leg raising test; no significant symptom relief after 1–3 months of conservative treatment; no spinal deformities; no abnormalities in liver or kidney function, and able to cooperate with follow-up work. Exclusion criteria: disc extrusion or free-floating protrusions; history of spinal surgery or existing spinal diseases; active infection or fever; bleeding tendency or taking medications affecting coagulation; severe mental health issues that prevent cooperation with follow-up. A follow-up visit was scheduled ninety days after treatment, and patients were divided into the Effective group (E group) and the Invalid group (I group) based on the modified Macnab criteria. Secondary treatment was conducted as needed, and a follow-up visit was scheduled again ninety days later.

### Treatment method

**Figure 1:**
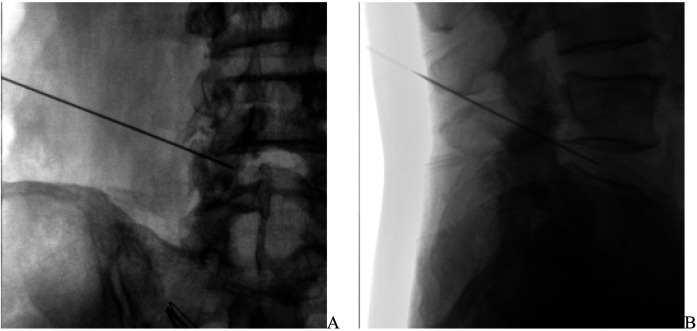
Under the guidance of the C-arm X-ray machine, the puncture needle reaches the target position in the intervertebral disc. **(A)** The position of the puncture needle on the anteroposterior view. **(B)** The position of the puncture needle on the lateral view.

All patients underwent lumbar disc radiofrequency ablation treatment guided by a C-arm x-ray machine. After the patients were admitted to the operating room, an intravenous line was established, and cardiac monitoring was connected. The patients lay prone on the operating table with a soft pillow under their abdomen to elevate the waist. After disinfection and draping, a needle was inserted 15 cm lateral to the target intervertebral disc, and the puncture was guided by the C-arm x-ray machine. Once the puncture needle reached the target position (Figure 1), the inner core was removed and the radiofrequency inner core was inserted. The radiofrequency device (Beijing Beiqi, model: R2000B) was connected, and both motor (2 Hz, 1.2 mV) and sensory (50 Hz, 1.2 mV) electrical stimulations were used to ensure the patient had no abnormalities. A 90°C continuous radiofrequency thermocoagulation was applied for 240 s. If the patient reported intense pain in the lower limbs during the treatment, the treatment was immediately stopped, the puncture needle position was adjusted, and the treatment was resumed. After the radiofrequency was completed, 2 ml of 0.5% ropivacaine was injected through the puncture needle, and the puncture site was covered with a sterile dressing. The patient was observed in the recovery room for 30 min without any abnormalities before being sent back to the ward.

### Observational indicators

After admission, record the basic information of the patients, including age, gender, body mass index, history of hypertension, history of diabetes, and history of coronary heart disease. On the morning of the second day after admission, collect a fasting blood sample, and use a blood cell analyzer to detect the absolute values of neutrophils and lymphocytes to calculate the Neutrophil-to-Lymphocyte Ratio (NLR), with repeat testing conducted 90 days and 180 days after treatment. Use the Visual Analogue Scale (VAS) for pain scoring, the Oswestry Disability Index (ODI) score, and the Japanese Orthopaedic Association (JOA) score to assess lumbar function in patients before treatment, 90 days after treatment, and 180 days after treatment.

### Statistical analysis

A follow-up visit was scheduled ninety days after treatment, and patients were divided into the Effective group (E group) and the Invalid group (I group) based on the modified Macnab criteria. Data analysis was conducted using the SPSS Statistics 23.0 software. The Shapiro–Wilk test was used to determine whether the data in each group were normally distributed. Normally distributed quantitative data were expressed as the mean ± standard deviation ($\bar{x}\pm s$), and intergroup comparisons were analyzed using independent samples *t*-tests. Intragroup comparisons were performed using repeated measures analysis of variance. Categorical data were expressed as medians and interquartile ranges, and numerical data were presented as counts and percentages. Categorical variables were analyzed using chi-square tests, continuity correction Chi-square tests, or Fisher's exact tests. Non-normally distributed quantitative data were expressed as medians (M) and interquartile ranges (IQR), and intergroup comparisons were made using the Mann–Whitney *U* test. Spearman's analysis was used to assess the correlation between preoperative NLR and postoperative ODI and JOA scores; ROC curve analysis was used to evaluate the predictive value of preoperative NLR for the outcomes of PIRFT treatment in patients with LDH. A *P*-value of less than 0.05 was considered statistically significant.

## Results

### Comparison of general conditions

**Figure 2:**
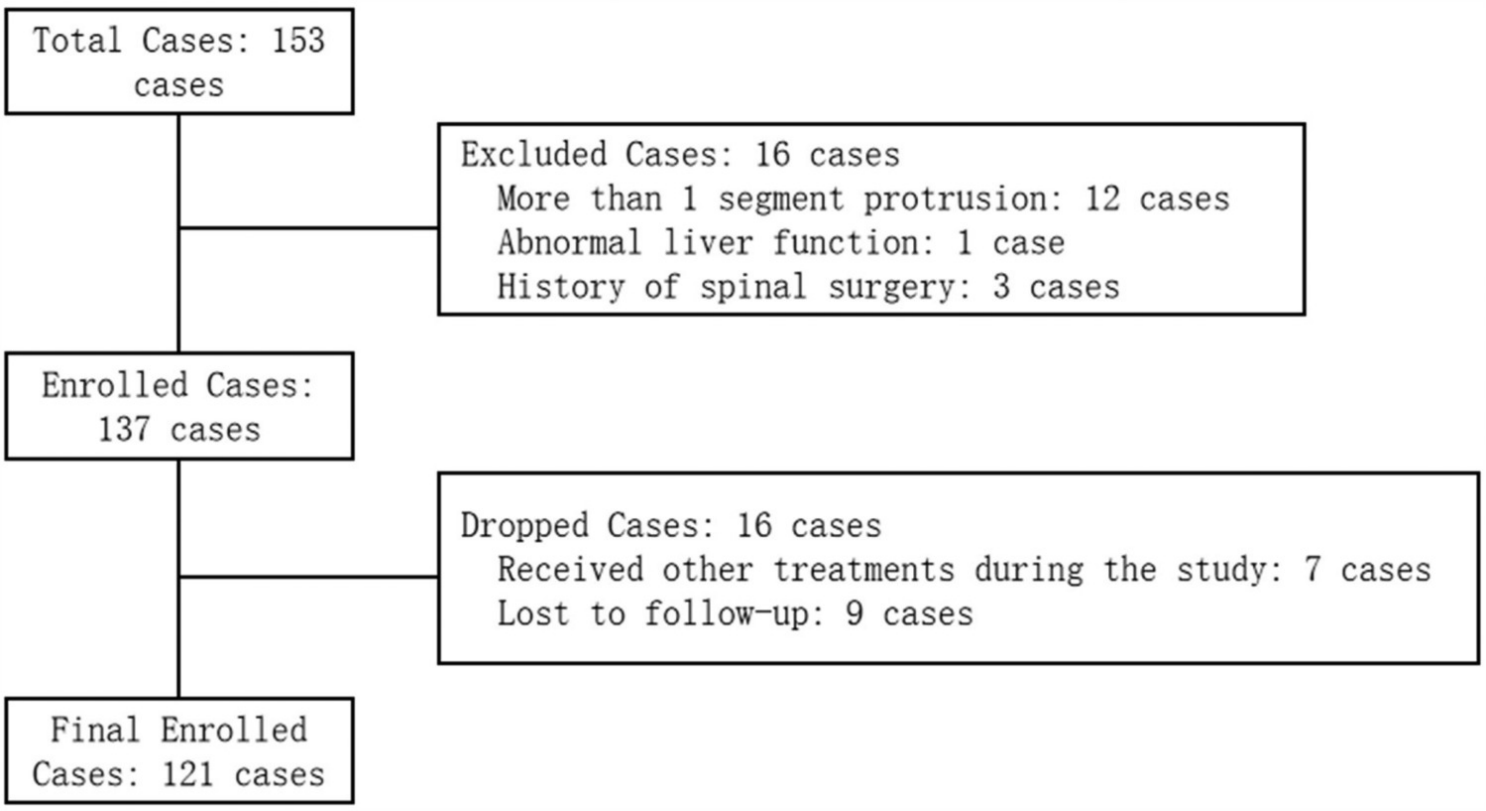
Patient Screening and Enrollment Flowchart.

From December 2023 to June 2024, our hospital's Pain Department received a total of 153 patients with lumbar disc herniation, all of whom underwent PIRFT treatment. In the preliminary screening phase, 16 patients were excluded either for not fulfilling the inclusion criteria or for meeting the exclusion criteria, leading to a total of 137 patients being enrolled. Throughout the study, 7 patients were excluded for undergoing treatments other than PIRFT, and 9 patients were excluded due to incomplete follow-up. Consequently, a total of 121 patients completed all study components and were included in the final analysis. The therapeutic effects after treatment were evaluated according to the modified Macnab criteria (10). We categorized Recovery, Significantly Effective, and Effective from the modified Macnab criteria into the Effective group (E group), and Invalid into the Invalid group (I group). The results showed that 110 patients were in the E group, and 11 patients were in the I group. There were no differences between the two groups in terms of age, gender, body mass index, protruding segments, and the presence of comorbidities such as hypertension, diabetes, coronary heart disease, and pulmonary diseases (*P* > 0.05) (Figure 2) (Table 1).

**Table 1:** Demographic and baseline characteristics.

Basic information	All patients (*n* = 121)	E group (*n* = 110)	I group (*n* = 11)	*t*/*χ*^2^	*P* value
Age	52.88 ± 7.51	52.76 ± 7.53	54.09 ± 7.50	0.557	0.578
Male	63	56	7	0.649	0.420
BMI	24.90 ± 2.31	24.81 ± 2.23	25.80 ± 2.98	1.361	0.176
Injured segment					
L_3−4_	18	17	1	1.161	0.560
L_4−5_	83	76	7		
L_5_-S_1_	20	17	3		
Smoking	31 (25.6%)	28 (25.5%)	3 (27.3%)	0.017	0.895
Alcohol	15 (12.4%)	15 (13.6%)	0 (0.0%)	1.712	0.191
Hypertension	35 (28.9%)	31 (28.2%)	4 (36.4%)	0.326	0.568
Diabetes	19	17	2	0.056	0.813
Cardiac Disease	8 (6.6%)	7 (6.4%)	1 (9.1%)	0.120	0.729
Pulmonary Disease	22 (18.2%)	22 (20.0%)	0 (0.0%)	2.689	0.101
Preoperative NLR levels	1.77 ± 0.43	1.71 ± 0.35	2.37 ± 0.70	3.094	0.011

### Patient treatment outcome statistics

After undergoing disc radiofrequency ablation treatment, 110 patients were found to have effective treatment results according to the modified Macnab criteria upon re-examination ninety days later, while 11 patients had poor treatment outcomes. These 11 patients all underwent PIRFT again, and upon re-examination ninety days later, their treatment effects were significantly improved (Table 2).

**Table 2:** Patient treatment outcome statistics.

Follow-up period	Effective (*n*)	Invalid (*n*)	*χ* ^2^	*P* value
90 days post-treatment	110	11	11.524	<0.001
180 days post-treatment	121	0		

### Comparison of NLR before and after treatment in both groups

**Figure 3:**
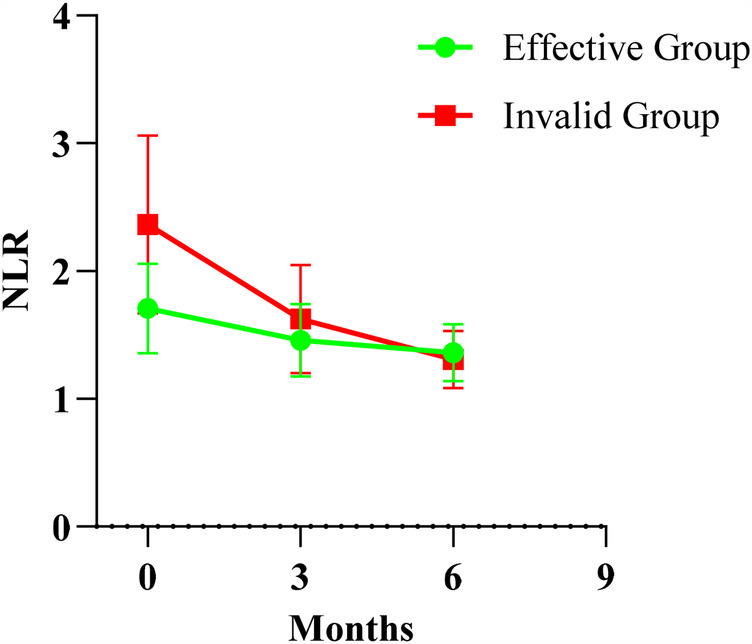
Comparison of NLR Levels at Various Time Points Between the Two Groups.

Intragroup and intergroup comparisons were made before and after treatment for both groups. The results showed that before treatment, the NLR level in the effective group was significantly lower than that in the ineffective group (*P* < 0.05). Ninety days after treatment, the NLR in both groups was lower than before treatment (*P* < 0.05), and at the same time, the NLR level in the effective group was lower than in the ineffective group (*P* < 0.05). One hundred and eighty days after treatment, the NLR levels in both groups were lower than before treatment and ninety days after treatment (*P* < 0.05), with no significant differences observed between the two groups (*P* > 0.05). (Figure 3).

#### Intergroup comparisons

Before treatment, comparison between E group and I group: *t* = 3.094, *P* = 0.011.

Ninety days after treatment, comparison between E group and I group: *t* = 1.822, *P* = 0.039.

One hundred and eighty days after treatment, comparison between E group and I group: *t* = 0.759, *P* = 0.449.

#### Intragroup comparisons

##### E group

Ninety days after treatment compared to before treatment: *t* = 2.199, *P* = 0.040.

One hundred and eighty days after treatment compared to before treatment: *t* = 8.696, *P* < 0.001.

One hundred and eighty days after treatment compared to ninety days after treatment: *t* = 2.075, *P* = 0.036.

#### I group

Ninety days after treatment compared to before treatment: *t* = 3.009, *P* = 0.007.

One hundred and eighty days after treatment compared to before treatment: *t* = 4.792, *P* < 0.001.

One hundred and eighty days after treatment compared to ninety days after treatment: *t* = 5.781, *P* < 0.001.

### Comparison of ODI scores before and after treatment in both groups

The Oswestry Disability Index (ODI) was used to evaluate the functional improvement of patients, consisting of 10 items that cover three major areas: pain (pain intensity, impact of pain on sleep), single functions (lifting, sitting, standing, walking), and personal comprehensive functions (daily living ability, sexual life, social activities, and outings) (11). The scores for each item are added together, with higher values indicating more severe functional disability.

**Figure 4:**
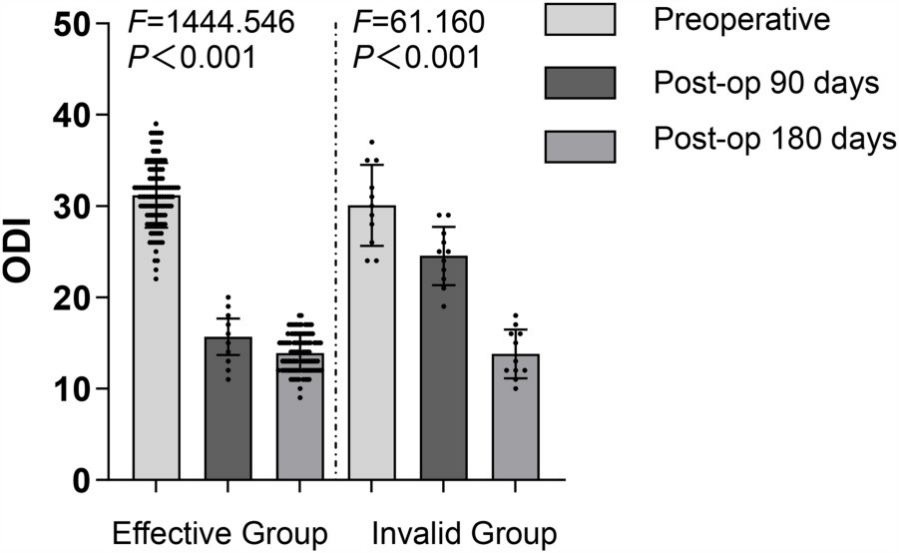
Comparison of ODI Scores at Various Time Points Between the Two Groups.

Before treatment, there was no significant difference in ODI scores between the two groups (*P* > 0.05). Ninety days after treatment, the ODI scores of E group were significantly better than those of I group (*P* < 0.05), while no significant difference was observed between the two groups one hundred and eighty days after treatment (*P* > 0.05). Within each group, the ODI scores ninety days after treatment were significantly lower than before treatment for both groups (*P* < 0.05), and the ODI scores one hundred and eighty days after treatment were significantly lower than before treatment and ninety days after treatment for both groups (*P* < 0.05) (Figure 4).

#### Intergroup comparisons

Before treatment, comparison between E group and I group: *t* = 0.965, *P* = 0.336;

Ninety days after treatment, comparison between E group and I group: *t* = 9.077, *P* < 0.001;

One hundred and eighty days after treatment, comparison between E group and I group: *t* = 0.137, *P* = 0.891;

#### Intragroup comparisons

##### E group

Ninety days after treatment compared to before treatment: *t* = 39.993, *P* < 0.001.

One hundred and eighty days after treatment compared to before treatment: *t* = 44.348, *P* < 0.001.

One hundred and eighty days after treatment compared to ninety days after treatment: *t* = 6.582, *P* < 0.001.

##### I group

Ninety days after treatment compared to before treatment: *t* = 3.371, *P* = 0.003.

One hundred and eighty days after treatment compared to before treatment: *t* = 10.415, *P* < 0.001.

One hundred and eighty days after treatment compared to ninety days after treatment: *t* = 8.570, *P* < 0.001.

### Comparison of JOA scores before and after treatment in both groups

The Japanese Orthopaedic Association Score (JOA) was used to assess the spinal function of patients, which includes subjective symptoms (low back pain, gait), clinical signs (straight leg raising test, sensory disturbance, motor disturbance), limitations in daily activities (lying flat and turning over, standing, walking, washing, etc.), and bladder function (12). Scores are categorized as follows: less than 10 points indicates poor condition; 10–15 points indicates good condition; 16–24 points indicates very good condition; 25–29 points indicates excellent condition.

**Figure 5:**
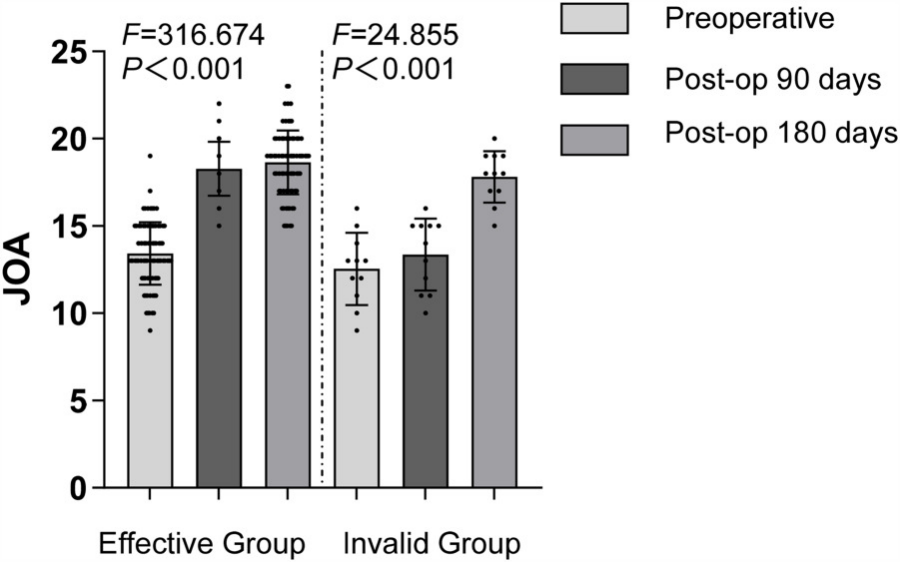
Comparison of JOA Scores at Various Time Points Between the Two Groups.

Comparison of JOA scores between the two groups before treatment showed no significant difference (*P* > 0.05). Ninety days after treatment, the JOA scores of the effective group were significantly better than those of the ineffective group (*P* < 0.05), and no significant difference was observed between the two groups one hundred and eighty days after treatment (*P* > 0.05). Within the effective group, JOA scores at three and one hundred and eighty days after treatment were significantly higher than before treatment (*P* < 0.05), and the score at one hundred and eighty days was higher than at ninety days after treatment (*P* < 0.05). In the ineffective group, the JOA score at ninety days after treatment showed no significant difference compared to before treatment (*P* > 0.05), but the score at one hundred and eighty days was significantly higher than before treatment and at ninety days after treatment (*P* < 0.05). (Figure 5).

#### Intergroup comparisons

Before treatment, comparison between E group and I group: *t* = 1.541, *P* = 0.126.

Ninety days after treatment, comparison between E group and I group: *t* = 9.815, *P* < 0.001.

One hundred and eighty days after treatment, comparison between E group and I group: *t* = 1.471, *P* = 0.144.

#### Intragroup comparisons

##### E group

Ninety days after treatment compared to: Before treatment *t* = 21.644, *P* < 0.001.

One hundred and eighty days after treatment compared to Before treatment: *t* = 21.484, *P* < 0.001.

One hundred and eighty days after treatment compared to ninety days after treatment: *t* = 1.640, *P* = 0.102.

##### I group

Ninety days after treatment compared to Before treatment: *t* = 0.929, *P* = 0.364.

One hundred and eighty days after treatment compared to Before treatment: *t* = 6.893, *P* < 0.001.

One hundred and eighty days after treatment compared to ninety days after treatment: *t* = 5.832, *P* < 0.001.

### Preoperative NLR levels correlation analysis with postoperative ODI and JOA scores

**Figure 6:**
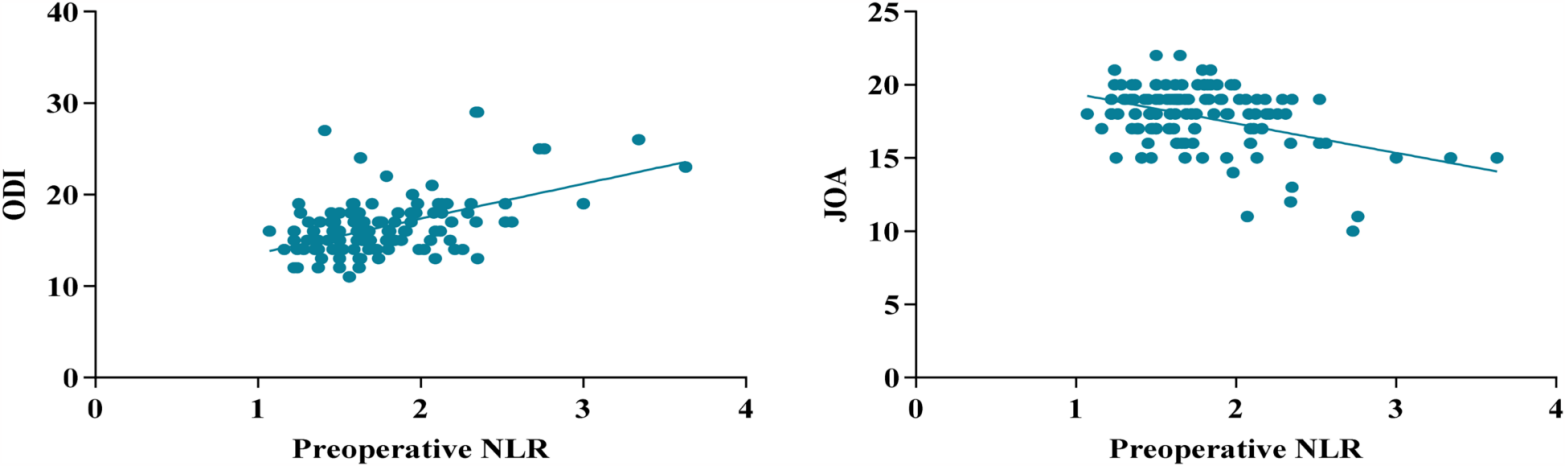
The correlation between preoperative NLR levels in patients with LDH and postoperative ODI and JOA scores.

Spearman's correlation analysis showed that the pre-treatment NLR levels in patients with LDH were positively correlated with the ODI scores at 90 days post-treatment, with *r* = 0.386, and *P* < 0.001; they were negatively correlated with the JOA scores, with *r* = −0.326 and *P* = 0.003. Due to 11 patients undergoing a second surgical procedure, the correlation between pre-treatment NLR levels and the 180-days post-treatment ODI and JOA scores was not analyzed (Figure 6).

### Receiver operating characteristic (ROC) curve

**Figure 7:**
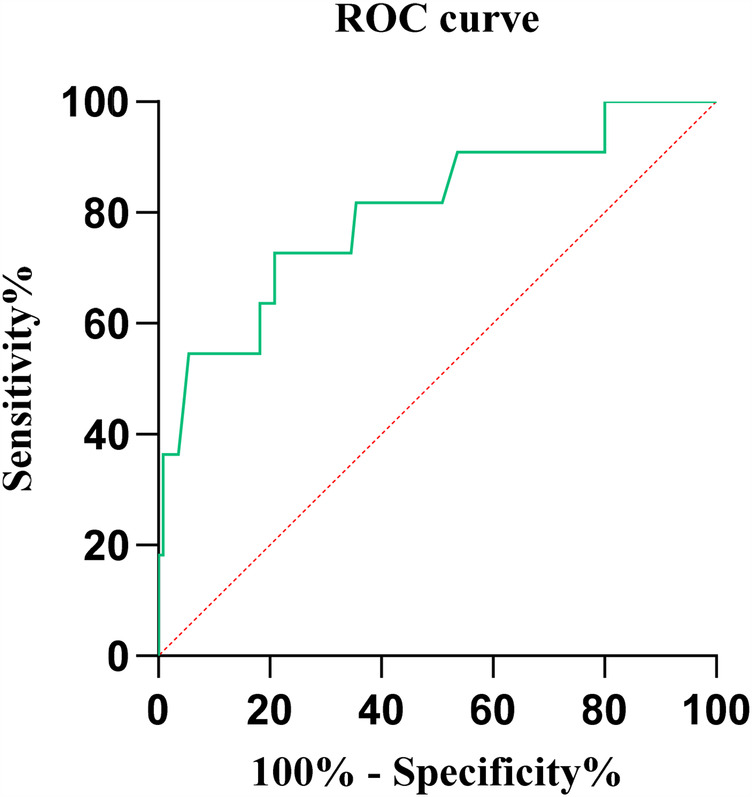
ROC curve analysis of the predictive value of preoperative NLR for treatment outcomes.

Using the ROC curve to explore the predictive ability of preoperative NLR for the efficacy of percutaneous disc radiofrequency ablation in the treatment of lumbar disc herniation, the results showed that the area under the ROC curve (AUC) was 0.803, the optimal diagnostic cutoff point was 1.975, the correct diagnosis index was 0.518, 95% CI: 0.650-0.955, *P* < 0.001 (Figure 7).

## Discussion

Low back and leg pain is extremely common in industrialized countries. According to research statistics (13), more than 30% of people will experience low back pain at least once in their lifetime. The causes of this pain are complex and varied, with about 85% being caused by lumbar disc herniation (LDH) (14). With the acceleration of modern life rhythms and changes in lifestyle, the incidence of LDH is increasing year by year, and the age of onset is becoming younger (1). Currently, the treatment methods for LDH mainly include two major categories: conservative treatment and surgical treatment. Although the treatment methods are different, the common goal of both is to relieve pain and restore the patient's mobility. Among many LDH patients, most have a small protrusion volume and are wrapped by the annulus fibrosus, which usually does not meet the criteria for traditional open surgical intervention. For these patients, percutaneous intradiscal radiofrequency thermocoagulation (PIRFT), as an advanced minimally invasive surgical technique, is undoubtedly an excellent treatment option. In clinical practice, PIRFT has shown significant therapeutic effects whether applied alone or in combination with other treatment methods (15, 16).

The compression of nerve roots is the main cause of pain in patients with lumbar disc herniation (LDH), and the accompanying inflammatory response activates and sensitizes the corresponding nociceptors, leading to a further expansion of the area of pain perception (17, 18). As a treatment method, percutaneous intradiscal radiofrequency thermocoagulation (PIRFT) effectively alleviates pain and promotes disc repair through various mechanisms, including reducing the volume of the protrusion, decreasing intradiscal pressure (19), ablating nerve endings within the annulus fibrosus, and regulating the expression of cytokines within the intervertebral disc (20). PIRFT modulates cytokine expression within the intervertebral disc via its thermal effect, decreasing the content of inflammatory mediators like Interleukin-1 (IL-1) and increasing the levels of Interleukin-8 (IL-8). Consequently, this approach ameliorates the intradiscal microenvironment and facilitates disc repair (21). In this study, the Oswestry Disability Index (ODI) score of patients in Group E was significantly lower after treatment compared to before treatment, and the Japanese Orthopaedic Association (JOA) score was significantly higher than before treatment; for Group I patients, the ODI score after treatment was significantly lower than before treatment, but the JOA score showed no significant difference compared to before treatment. This indicates that the PIRFT treatment method can indeed effectively reduce patient pain and significantly improve their activity ability. Regarding the non-significant difference in JOA scores for Group I patients after treatment compared to before treatment, the analysis suggests that it may be related to higher levels of inflammation in the body, meaning that inflammatory responses may pose certain obstacles to the recovery of muscle strength. This is similar to the results of a previous study, where NORHEIM and colleagues found in a study of 214 elderly patients (22) that during hospitalization, those showing signs of inflammation often had more limited improvements in mobility compared to those without signs of inflammation.

In recent years, with in-depth research on the pathophysiological mechanisms of lumbar disc herniation (LDH), an increasing amount of evidence suggests that the inflammatory response in the peripheral nervous system plays a crucial role in the development and persistence of pathological pain states triggered by LDH (23, 24). When a disc herniation occurs, its contents, such as the nucleus pulposus, are perceived as foreign by the immune system, leading to an inflammatory response (25). Inflammatory cells, such as neutrophils and macrophages, are drawn to the site of the herniation and release cytokines including Tumor Necrosis Factor-alpha (TNF-α), Interleukin-6 (IL-6), Interleukin-1 beta(IL-1β), and Prostaglandin E2 (PGE2), which amplify the inflammatory process (26). Monocyte Chemoattractant Protein-1 (MCP-1), a chemokine produced by disc and inflammatory cells, facilitates the directed movement of neutrophils to the site of inflammation (27). Together, these factors contribute to the formation of a complex neuro-immune interaction network, resulting in the pathophysiological changes characteristic of discogenic low back pain. Therefore, reducing inflammation has become a new direction and research focus in the treatment of LDH. Controlling inflammation as a key link in the treatment of LDH, and through the early identification of inflammation levels and the adoption of individualized intervention measures, it is expected to significantly improve treatment effects and accelerate the speed of recovery. Neutrophils and lymphocytes are important components of the immune system. Neutrophils usually increase in acute inflammatory responses, while lymphocytes may decrease in chronic inflammation or immunosuppressed states. Several studies have confirmed the value of the Neutrophil-to-Lymphocyte Ratio (NLR) in the prognosis evaluation of inflammatory diseases, showing high sensitivity and specificity in reflecting systemic inflammatory states (8, 28). Moreover, calculating NLR only requires two parameters from routine blood tests, and this simple, non-invasive, and cost-effective testing method is widely applied in clinical practice. In light of this, the aim of this study is to explore whether NLR can serve as an effective tool for predicting the therapeutic effects of patients with LDH undergoing PIRFT. In this study, we observed that the NLR of patients in both groups significantly decreased after PIRFT treatment. The reduction of NLR is generally considered an indicator of reduced inflammatory responses in the body, and this finding strongly supports the effectiveness of the PIRFT treatment method in reducing the release of inflammatory mediators. Combined with the significant reduction in ODI scores after treatment, we can conclude that the significant improvement in patients’ pain symptoms and functional disabilities is highly consistent with the reduction of inflammatory responses in the body. Spearman's correlation analysis showed that the pre-treatment NLR levels in patients with LDH were positively correlated with the ODI scores at 90 days post-treatment and negatively correlated with the JOA scores. This finding indicates that higher pre-treatment NLR levels may predict poorer treatment responses and slower functional recovery, which is of significant clinical importance. In summary, pre-treatment NLR has certain reference value in predicting the effects of PIRFT treatment for LDH, and patients with poor postoperative mobility recovery often have higher preoperative NLR values. A higher preoperative NLR ratio can serve as an important reference indicator, helping doctors to consider anti-inflammatory treatment before deciding whether to use PIRFT treatment, in order to improve therapeutic effects.

Despite the achievements of this study, there are some undeniable limitations. First, due to the small sample size and short follow-up period, the results may be biased, which could affect the generalizability of the conclusions. Second, when selecting patients for this study, we only recorded the presence or absence of pulmonary diseases, neglecting the coexistence of other systemic inflammations. These comorbidities, which were not fully considered, may have complex effects on the NLR ratio and its prognosis for surgical treatment outcomes. Furthermore, although we confirmed the impact of inflammatory responses on the postoperative recovery of LDH patients, we were unable to collect preoperative and postoperative inflammatory cytokine data from the patients, preventing us from accurately determining their specific changes at the cytokine level. This aspect also needs to be further explored and clarified in subsequent studies to provide more precise guidance for clinical practice.

## Conclusion

This study reveals the correlation between high neutrophil-to-lymphocyte ratio (NLR) and the recovery of patients with lumbar disc herniation (LDH) after percutaneous intradiscal radiofrequency thermocoagulation (PIRFT) treatment. In-depth analysis indicates that the inflammatory marker NLR has some reference value in predicting the effectiveness of PIRFT treatment for LDH. Therefore, managing inflammation levels should be considered a key objective in the treatment process for LDH patients. Medical professionals should adopt more meticulous and cautious treatment strategies when facing patients with high preoperative NLR levels. In summary, as an economical, widely available, and easily assessable peripheral blood marker, NLR has the potential to become an effective indicator for predicting postoperative recovery in LDH patients. However, to further validate our findings and explore the role and mechanisms of NLR in the postoperative recovery of LDH patients, more research is needed, especially large-scale clinical studies.

## Data Availability

The datasets presented in this study can be found in online repositories. The names of the repository/repositories and accession number(s) can be found below: http://www.medresman.org.cn.
